# Quality assessment of an interferon-gamma release assay for tuberculosis infection in a resource-limited setting

**DOI:** 10.1186/1471-2334-9-66

**Published:** 2009-05-18

**Authors:** Nguyen TL Hang, Naoki Ishizuka, Naoto Keicho, Le T Hong, Do B Tam, Vu TX Thu, Ikumi Matsushita, Nobuyuki Harada, Kazue Higuchi, Shinsaku Sakurada, Luu T Lien

**Affiliations:** 1 IMCJ-BMH Medical Collaboration Center, Bach Mai Hospital, Hanoi, Viet Nam; 2 International Medical Center of Japan, Research Institute, Tokyo, Japan; 3 Hanoi Tuberculosis and Lung Disease Hospital, Hanoi, Viet Nam; 4 Research Institute of Tuberculosis, Japan Anti-Tuberculosis Association, Tokyo, Japan

## Abstract

**Background:**

When a test for diagnosis of infectious diseases is introduced in a resource-limited setting, monitoring quality is a major concern. An optimized design of experiment and statistical models are required for this assessment.

**Methods:**

Interferon-gamma release assay to detect tuberculosis (TB) infection from whole blood was tested in Hanoi, Viet Nam. Balanced incomplete block design (BIBD) was planned and fixed-effect models with heterogeneous error variance were used for analysis. In the first trial, the whole blood from 12 donors was incubated with nil, TB-specific antigens or mitogen. In 72 measurements, two laboratory members exchanged their roles in harvesting plasma and testing for interferon-gamma release using enzyme linked immunosorbent assay (ELISA) technique. After intervention including checkup of all steps and standard operation procedures, the second trial was implemented in a similar manner.

**Results:**

The lack of precision in the first trial was clearly demonstrated. Large within-individual error was significantly affected by both harvester and ELISA operator, indicating that both of the steps had problems. After the intervention, overall within-individual error was significantly reduced (*P *< 0.0001) and error variance was no longer affected by laboratory personnel in charge, indicating that a marked improvement could be objectively observed.

**Conclusion:**

BIBD and analysis of fixed-effect models with heterogeneous variance are suitable and useful for objective and individualized assessment of proficiency in a multistep diagnostic test for infectious diseases in a resource-constrained laboratory. The action plan based on our findings would be worth considering when monitoring for internal quality control is difficult on site.

## Background

Assuring quality is essential for clinical laboratories in the field of infectious diseases. Beneficiaries are not only patients obtaining a diagnosis on site but also future patients receiving benefits of clinical research supported by qualified laboratories. Quality assurance in modern laboratories is realized by total quality management including external quality assurance (EQA) and internal quality control (IQC) [1-3].

In most resource-constrained countries, however, regulations on quality assurance have not been laid down by the authorities and accuracy and precision of clinical measurements have not been monitored systematically [4]. Under such disadvantageous circumstances, when important but rather complicated testing for infectious diseases is undertaken, we cannot easily be confident that the skill has been transferred and maintained properly until the procedure becomes familiar and stably performed in accordance with a desirable quality control system [5]. During this vulnerable period, how to assess proficiency of the testing effectively and objectively, and how to assure and improve the quality are open issues to be addressed.

Currently, immunoassay is commonly used to make a serological diagnosis of infectious diseases involving human immunodeficiency virus, a variety of hepatitis virus and other sexually transmitted or blood-borne pathogens [6,7], which are serious problems in the developing world. Enzyme linked immunosorbent assay (ELISA) is often used to make diagnosis of these diseases in the clinical laboratories. Because of the complexity of the method, however, quality control of these assay systems is challenging [8]. In this context, trend of point of care (POC) tests that facilitate immediate and on-site diagnosis as well as early treatment of infectious diseases has been emphasized [7]. However, their usage in resource-constrained countries is still hampered by high cost and difficulties in testing for high throughput screening and thus laboratory-based immunoassays would be irreplaceable in many fields.

Recently, a two-step immunoassay to detect tuberculosis (TB) infection has also been developed and used extensively [9]. It consists of whole blood stimulation with TB-specific antigens followed by measurement of interferon-gamma using ELISA. Our objective in the present study is to demonstrate that the quality of laboratory tests can be assessed objectively even in a resource-constrained laboratory if the optimum design of experiments and appropriate statistical models are chosen. As a result of this attempt, we experienced marked improvement of the quality of this multi-step immunoassay made by more than one laboratory staff member in a hospital of Viet Nam. We proposed a general plan to evaluate skills of laboratory staff members efficiently and quantitatively to perform qualified immuno-diagnostic testing especially for infectious diseases until such time as they establish a total quality management system by themselves.

## Methods

### Interferon-gamma release assay (IGRA) for diagnosis of TB infection

IGRA is a general method to measure interferon-gamma induced by *Mycobacterium tuberculosis*-specific antigens (TB-Antigen) for detecting TB infection. In the ELISA-based IGRA (QuantiFERON-TB Gold In-Tube™, Cellestis, Victoria, Australia), one milliliter of the whole blood was collected into the Nil tube for negative control, Mitogen for positive control, and TB-Antigen separately. The blood in the tubes was mixed and placed in the incubator for 18 hours at 37°C (Cool incubator NC-25B, Funakoshi, Tokyo, Japan). Approximately 200 μl of plasma were harvested from each tube after centrifugation (Kubota 2010, Kubota, Tokyo, Japan).

Interferon-gamma concentrations in the plasma were measured by ELISA, using microtiter plate washer and reader (Wellwash Plus Microplate Washer and Multiscan JX Microplate Reader, Thermo Electron Corporation, Vantaa, Finland) with the analysis software provided by the manufacturer (QuantiFERON-TB Gold Analysis Software, ver. 2.50, Cellestis). In this study, interferon-gamma concentrations obtained from this calculation were directly used for further analysis.

### Study setting

Two trials were carried out in Hanoi TB and Lung Disease Hospital, Viet Nam. Between the first and second trial, statistical analysis was made and an intervention was planned to ensure counterchecking and correct questionable manipulations. Each trial consisted of two runs. In each run, three milliliters of blood were collected from volunteers after informed consent had been obtained. Study protocols using IGRA were approved by ethical committees of the Ministry of Health, Viet Nam and International Medical Center of Japan respectively.

Two laboratory members, A and B, performed either plasma harvest or ELISA operation or both: Harvest included labeling and placing plasma storage tubes properly and transferring plasma from centrifuged blood collection tubes to these tubes by pipetting. ELISA was a process including preparing reagents and transferring plasma samples into the microtiter plate. ELISA ended with calculation of interferon-gamma concentration. Because their roles were changed occasionally due to the limited manpower of the laboratory, their performance in both Harvest and ELISA was the subject to be analyzed.

### Balanced incomplete block design (BIBD)

A single specimen obtained from routine blood collection was not sufficient to assess two staff members' performance. Because additional blood sampling was not easily accepted in many countries including Viet Nam, BIBD was attempted to obtain analytical information from small volume of plasma samples in this study: Of four possible combinations of harvester and ELISA operator, two combinations were cyclically chosen, using the limited amount of specimen. Allocation of observed combinations by BIBD in this study was described in Table 1. In each trial, there were two levels of Harvest (two different Harvesters), two levels of ELISA (two different ELISA operators) and 12 levels of Specimen (12 different blood donors).

**Table 1:** Allocation of observed combinations of Harvester and ELISA operator.

Sample	Specimen*	Harvest	ELISA	Data	Sample	Specimen	Harvest	ELISA	Data
	
1	1	A	A	Observed	7	4	A	A	Observed
2	1	A	B	Observed		4	A	B	Not observed
	1	B	A	Not observed	8	4	B	A	Observed
	1	B	B	Not observed		4	B	B	Not observed
	2	A	A	Not observed		5	A	A	Not observed
	2	A	B	Not observed	9	5	A	B	Observed
3	2	B	A	Observed		5	B	A	Not observed
4	2	B	B	Observed	10	5	B	B	Observed
5	3	A	A	Observed		6	A	A	Not observed
	3	A	B	Not observed	11	6	A	B	Observed
	3	B	A	Not observed	12	6	B	A	Observed
6	3	B	B	Observed		6	B	B	Not observed

*To each specimen, two measurements were assigned. This layout was repeated twice by using different sets of specimens in each trial.

### Outliers

To identify outliers, Mahalanobis distance D was calculated, which took the distance from the mean and correlation into account [10]. When D > 2.0, the value of that observed pair was regarded as outlier.

### A fixed-effect model and three-way analysis of variance (ANOVA)

To assess effects of factors of interest and error variance, we used a fixed-effect model:

of which,

y_ijk_: Interferon-gamma concentration in the plasma

μ: Grand mean of all measurements

α_i_: Harvest with i levels: i = 1, 2 (= A and B)

β_j_: ELISA with j levels: j = 1, 2 (= A and B)

γ_k_: Blood specimen with k levels: k = 1, 2,..., 12

ε_ijk_: Within-individual error; following normal distribution with mean = 0 and variance = σ^2^: N (0, σ^2^)

In this clinical setting, effects of interaction terms were not considered in the above model, because harvesting plasma and performing ELISA are independent steps and it is unlikely that the exchanging of staff roles in itself could increase the chances of error.

### Analysis of heterogeneous error variance affected by a given factor

To determine whether individuals of Harvest or ELISA affect within-individual error, we assessed a fixed-effect model with heterogeneous variance of error in the following way:

where error follows the normal distribution N(0, σ_ij_^2^).

Error variance affected by Harvesters was evaluated in the following formula:

Similarly, the following formula was used for error variance affected by ELISA operators:

### Coefficient of variation (CV) before and after intervention

Error variance ε_ijk _that included sources of Harvest and ELISA was calculated in a simple one-way ANOVA model adjusted by specimen. Based on the following formula, CVs of the two trials were assessed:

CV should not be larger than 20% in any types of immunoassay [8].

### Assessment of heterogeneous variance between the two trials

To analyze overall within-individual error between the two trials statistically, we used a fixed-effect model with heterogeneous variance of error, under the assumption that α and β were fixed throughout the trials. The effect of each blood specimen γ was expected to be different between the two trials.

of which, ε_ijk1 _and ε_ijk2 _were within-individual errors of the first and the second trial respectively. On the above assumption, ε_ijk1 _and ε_ijk2 _would be heterogeneous error between the trials.

Calculation of Mahalanobis distance, three-way ANOVA and estimation of heterogeneous variance were performed by SAS version 9.1 (SAS Institute Cary, NC, USA). Differences in error variance of two trials and error variance affected by a given factor were considered to be significant when *P*-value was less than 0.05.

## Results

### Evaluation of outliers and three-way ANOVA in the first trial

Out of 72 measurements obtained from the first trial, seven outliers were identified: One was in Nil condition, three in TB-Antigen and three in Mitogen (Mahalanobis D = 2.64 to 4.69).

To assess effects of individuals for Harvest and ELISA and character of errors involved in the first trial, we first performed three-way ANOVA using a fixed-effect model, in which three factors, Harvest, ELISA and individual blood specimens may have possible effects on the interferon-gamma concentration respectively. This model decomposes the total variance into between-individual error (or bias) and within-individual error (or imprecision). Herein, "between-individual error" indicates deviation in interferon-gamma values caused by the difference between Harvesters or ELISA operators, and "within-individual error" represents fluctuation of interferon-gamma values measured by a single Harvester or ELISA operator.

As shown in Table 2, mean square error indicating magnitude of within-individual error was large in all conditions of the first trial, which was indicated by remarkably large CV (> 20%) for Nil, TB-Antigen and Mitogen. Furthermore, in the condition of Mitogen, the mean-square value directing the effect of ELISA, or "between-individual error", was significantly large (*P *= 0.017). In the other two conditions, the effects of ELISA and Harvest were also considerably large but did not reach significant levels, as compared with the corresponding mean square errors. These findings indicate that their performance is unstable. Problems specific to ELISA and Harvest should be considered, although not statistically significant in all conditions.

**Table 2:** Three-way analysis of variance in the first trial.

	Nil	TB-Antigen	Mitogen
Mean (IU/ml)	0.7821	5.4013	15.3638
Harvest			
Mean Square	0.0000	1.5252	51.2656
F value	0.0000	0.2200	2.5600
*P *value	0.9984	0.6482	0.1404
ELISA			
Mean Square	1.8838	12.3026	161.3535
F value	1.2600	1.7800	8.0700
*P *value	0.2847	0.2112	0.0175
Error			
Mean Square	1.4741	6.8935	19.9916
Root Mean Square	1.2142	2.6255	4.4712
Coefficient of Variation (%)	155.2476	48.6099	29.1022

### Analysis of heterogeneous error variance in the first trial

We then analyzed which factor affected within-individual error. Because two laboratory members were involved in each step of this experiment, we assumed that within-individual error, i.e. error variance, could be different depending on the personnel in each step. Thus, we chose a fixed-effect model with heterogeneous variance of error affected by Harvest and ELISA (Table 3).

**Table 3:** Error variance affected by Harvester (left) and error variance affected by ELISA operator (right) in the first trial.

ε*i*.	Harvester	*P *value	ε*.j*	ELISA operator	*P *value
	
Nil	A:1.9150 B:0.0160	0.0040	Nil	A:0.0036 B:3.2723	0.0244
TB-Antigen	A:2.9897 B:9.7114	0.2546	TB-Antigen	A: 0.1270 B:15.2216	0.0830
Mitogen	A:33.5782 B: 5.6792	0.2780	Mitogen	A:41.1154 B: 3.0221	0.3584

In Nil condition, difference in error variance was statistically significant between Harvesters A and B (*P *= 0.0040), when error variance caused by ELISA operator was not considered. Difference of error variance caused by ELISA operators A and B was also significant (*P *= 0.024), when error variance caused by Harvester was not taken into account. These findings imply that under the model, the error variance was affected significantly by different Harvesters or ELISA operators, respectively.

### Intervention

By means of the above-mentioned statistical analysis of the first trial, we identified several points to be improved: a) there was a considerable number of outliers. Within-individual error was large and between-individual error was also comparably large, and b) within-individual error was affected by both Harvesters and ELISA operators at least when Nil was measured.

Based on these results, an intervention was introduced: 1) reviewing all procedures of Harvest and ELISA, 2) reconsidering and strengthening standard operation procedures, 3) checking working condition of machines, and 4) developing a checklist for countercheck. First, we attempted to find out which procedure of harvesting and ELISA operation would be unstable and all questionable manipulations were listed up. Essential laboratory skills, such as mixing the solution by pipetting, were reviewed. Secondly, standard operation procedures were rechecked and corrected seeing that the laboratory personnel were handling three blood collection tubes and three other plasma storage tubes from each blood donor at a time, they should take every care to identify the tubes during Harvest and ELISA and to confirm the right position of corresponding tubes. Thorough instruction for handling ELISA plates and tubes with manipulation of the pipette was given to avoid carry-over error or contamination. After intensive discussions, more attention was paid to basic laboratory practice and reduction of preventable mistakes. Thirdly, performance of the ELISA plate washer and reader and the quality of distilled water were also checked. Technical requirements from the manufacturer, such as temperature for reagent reservation, time of incubation, were strictly followed. Finally, a checklist for the countercheck of each step was developed for practical use.

### General assessment by CV before and after intervention

To assess the overall improvement after intervention, CV was compared between the two trials. Because variation due to Harvest and ELISA was of interest, CV adjusted by the effect of specimens was calculated and used. The CV had decreased remarkably in each condition of the second trial, as compared with that of the first trial, indicating the overall improvement of test performance after intervention (Table 4).

**Table 4:** CV adjusted by specimen in the two trials.

	CV (%)	
	
Condition	1^st ^trial	2^nd ^trial
Nil	150.5036	2.1661
TB-Antigen	48.6219	2.3967
Mitogen	38.1630	9.8776

### Evaluation of outliers and three-way ANOVA in the second trial

In the second trial, only one outlier was seen in Nil condition (Mahalanobis D = 2.59); the number of outliers was lower than that of the first trial.

**Figure 1:**
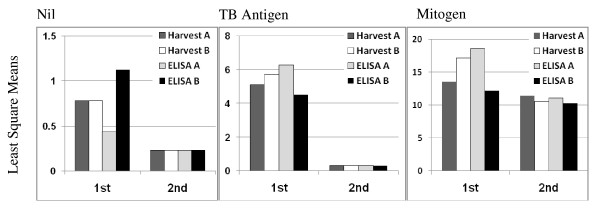
**Least square means of measurements in the first and the second trials**. Differences in least square means between Harvesters and between ELISA operators in the conditions of Nil, TB-Antigen and Mitogen in the second trial were compared with those in the first trial.

We then proceeded to analyze the change of parameters that had possibly contributed to overall improvement of test performance. As shown in Table 5, both mean square error and mean-square values showing effects of Harvest and of ELISA were markedly lower in the second trial. The former implies the decrease in within-individual error and the latter shows the reduction of between-individual error. The latter change was also clearly shown when differences of least square means between Harvesters and between ELISA operators in each condition of the second trial were compared with those in the first trial (Figure 1).

**Table 5:** Three-way analysis of variance in the second trial.

	Nil	TB-Antigen	Mitogen
Mean (IU/ml)	0.2308	0.3071	11.0017
Harvest			
Mean Square	0.0000	0.0000	3.4225
F value	1.0000	0.1000	3.2100
*P* value	0.3409	0.7572	0.1036
ELISA			
Mean Square	0.0000	0.0000	0.0770
F value	1.0000	0.4000	0.0700
*P* value	0.3409	0.5393	0.7937
Error			
Mean Square	0.0000	0.0001	1.1707
Root Mean Square	0.0050	0.0079	1.0330
Coefficient of Variation (%)	2.1661	2.5615	9.3898

### Analysis of heterogeneous error variance affected by harvester and ELISA operator in the second trial

In contrast to the first trial, there were no significant differences of error variance affected by Harvesters or ELISA operators (Table 6). This finding showed that the heterogeneous error variance indicating personnel-dependent unstableness was small enough in each step of the second trial.

**Table 6:** Error variance affected by Harvester (left) and error variance affected by ELISA operator (right) in the second trial.

ε*i*.	Harvester	*P *value	ε.*j*	ELISA operator	*P *value
	
Nil	NE* (A > B)		Nil	A:0.000014 B:0.000035	0.4832
TB-Antigen	A:0.0001 B:0.00002	0.2391	TB-Antigen	NE (A > B)	
Mitogen	A:2.1184 B: 0.1788	0.3291	Mitogen	A:1.9065 B: 0.2747	0.33347

*NE = not estimable by this calculation.

### Assessment of heterogeneous variance between the two trials

We further evaluated the decrease in overall within-individual error statistically. For this purpose, we used a fixed-effect model with heterogeneous variance between the two trials. Under the assumption that influence of Harvest and ELISA was not changed between the two trials, estimated overall error variances of the two trials were compared. As shown in Table 7, values indicating the overall within-individual error in all conditions had significantly decreased in the second trial (*P *< 0.0001).

**Table 7:** Difference in estimated overall within-individual error between the two trials.

	Estimated overall error variance (ε_ijk_)	
	
Condition	1st trial	2nd trial*
Nil	1.3866	0.000025
TB-Antigen	6.9003	0.000062
Mitogen	35.3152	1.0814

*P < 0.0001

## Discussion

We have demonstrated that a study design BIBD and statistical analysis using fixed-effect models with heterogeneous variance of error are useful for objective and quantitative assessment of laboratory testing for the first time. A series of experiments in our study clearly showed that proficiency of the personnel was improved by an appropriate intervention between the first and second trials of a two-step ELISA-based immunoassay for tuberculosis newly introduced to a resource-constrained laboratory.

Design of clinical experiments including block designs can be used to estimate effect of factors and their possible interaction [10]. In block designs including BIBD, introduction of blocks usually provides extra precision for comparison of other factors, while difference between blocks is of no intrinsic interest [10]. In our proficiency testing, variation of individual specimens was not the point of interest, but analysis of the other two factors, Harvest and ELISA was of importance. Roles of laboratory members are occasionally changed because of limited manpower. In such a case, our analysis is indispensable for assessment of their individual skills in each step of the testing, since this kind of approach has not been evaluated by the conventional IQC methods [11].

Previous studies in clinical fields other than laboratory medicine showed the advantage of BIBD over the sample size [12-14]. In the present study, this design enabled us to evaluate essential components of the blood testing procedure systematically without collecting an extra specimen from each donor. If all combinations of Harvesters and ELISA operators were to be tested at the same time, a twice-larger volume of blood should be collected from each volunteer, however, obtaining consent of this often causes difficulties in a country where blood sampling is not easily accepted. We have shown furthermore that this design is suitable for clinical settings in which many different specimens are to be handled at the same time.

In the first trial before intervention, we found that within-individual error was large and between-individual error tended to be so. However, a number of outliers also affected both within- and between-individual errors. The cause of outliers was probably due to mixing up of specimen tubes or contamination of samples resulting from unfamiliar handling of multiple samples, although this was not easily determined [15,16]. Using a fixed-effect model with the heterogeneity of error variance, we further illustrated that within-individual error was affected by Harvesters and ELISA operators. The results indicated that there were problems with both steps of Harvest and ELISA, and with both laboratory members, and this represented a strong motivation to improve the skills of the laboratory personnel in both steps of Harvest and ELISA.

After timely intervention including checkup of all steps and standard operation procedures, marked improvement was observed in all parameters including CV, a general parameter for precision of measurements [8]. In case of IGRA in this study, CV should be kept less than 10% [17,18] and in the second trial, this criterion was met satisfactorily.

We propose as a consequence the following action plan to improve diagnostic capacity in resource-constrained settings. This could be generalized not only for complicated immunoassay for infectious disease but also for other kinds of clinical tests:

• Set the target CV derived from simple one-way ANOVA model of specimen (for example, 10%). This value should be defined before the commencement of study.

• Design experiment to evaluate between- and within-individual error.

• Conduct experiment.

• Analyze data with ANOVA model with and without heterogeneous error variance.

• If CV exceeds the target, review the operating procedures.

• Conduct experiment a second time.

• Consequently analyze data to ascertain any improvement. 

• Return to step 5 until CV becomes less than the target.

In-house quality control for effective transfer of skills is a topic of interest in our proposal and this should be carried out easily, at a low cost, whilst assuring objective and quantitative assessment in a clinical laboratory where resources such as reagents, manpower and feasibility of sample collection are limited. Our plan meets the above requirement. Measurements could be sent via the internet and analyzed in a statistical way by a joint-research facility inside or outside the country and an immediate feedback should be sent in an appropriate manner. Such continuous efforts to share information are important to maintain quality levels over a long distance [19].

In this age of evidence-based medicine and development of new diagnostic technologies, quality of laboratory tests is essential. There is an urgent need for validation and standardization of the new assays before they are adopted into clinical diagnostics [20]. Until such time as an effective quality control system is established, our approach is valuable to assure the quality of laboratory tests for timely diagnosis and treatment of infectious diseases. Another favorable design or analytical method might be suggested by others in the future studies, seeing that no standard way of quality monitoring has been proposed so far. We expect that the successful experience gathered in the present study will provide useful information for further comparison and discussion.

Our study has some limitations. It was obvious that outliers influenced statistical analysis in the first trial and exact causes of error in each condition were not clearly specified by the present analysis itself [15]. Through repeated experiments, the causes of error might be clearer, although all errors in our study decreased dramatically after a single intervention. We should also emphasize in conclusion, that a number of procedures should be combined to establish a total quality assurance system.

## Conclusion

In a setting where a modern quality control system has not been entirely established, a laboratory test could be assessed quantitatively and such objective assessment is helpful for quality improvement of the test, if an appropriate design of experiment and statistical method are chosen. The design of experiment BIBD and analytical models for ANOVA were useful for objective assessment of individual skills in each stage of a multi-step immunoassay for tuberculosis in a laboratory with limited resources. A proposed plan to assess the level of proficiency might be useful for skill improvement of clinical testing especially for infectious diseases when monitoring is difficult to assure the sustainability of the technology transferred.

## Competing interests

The authors declare that they have no competing interests.

## Authors' contributions

NTLH participated in supervising the on-site implementation of the study, drafting the paper or substantially revising it. NI was responsible for making conception, design and overall supervision of the study, analysis and interpretation of data, drafting the paper or substantially revising it. NK participated in making conception and design of the study, analysis and interpretation of data, drafting the paper or substantially revising it. LTH carried out the immunoassays. DBT participated in on-site implementation. VTXT carried out the immunoassays. IM participated in technical transfer and supervision. NH was responsible for technical transfer and supervision. KH was responsible for technical transfer and supervision. SS participated in conception and design of the study. LTL participated in conception, design and supervision of the study. All authors read and approved the manuscript.

## Pre-publication history

The pre-publication history for this paper can be accessed here:

http://www.biomedcentral.com/1471-2334/9/66/prepub
